# The Interplay of Quality of Life and Satisfaction with Nursing Care in Cancer Patients: A Narrative Review

**DOI:** 10.3390/clinpract16080140

**Published:** 2026-07-27

**Authors:** Efthymia Vlachothanasi, Ioanna Tsatsou, Theocharis I. Konstantinidis, Maria Saridi, Evangelos C. Fradelos, Georgios Goumas, Pavlos Sarafis

**Affiliations:** 1 Department of Nursing, University of Thessaly, 41500 Larissa, Greece; efoula12@gmail.com (E.V.); msaridi@uth.gr (M.S.); efradelos@uth.gr (E.C.F.); psarafis@uth.gr (P.S.); 2 Department of Nursing, University of West Attica, 12243 Egaleo, Greece; 3 Department of Nursing, Hellenic Mediterranean University, 71410 Heraklion, Greece; harriskon@hmu.gr; 4 School of Public Health, University of West Attica, 11521 Athens, Greece; ggoumas@uniwa.gr

**Keywords:** oncology nursing care, cancer nursing, cancer patients, quality of life (QoL), satisfaction with nursing care (SNC), patient-reported outcomes, conceptual frameworks

## Abstract

This narrative review explores the interconnected relationship between quality of life (QoL) and satisfaction with nursing care (SNC) among cancer patients, recognizing that cancer’s burden extends beyond survival and significantly affects physical, psychological, and social well-being. A non-systematic search of PubMed, CINAHL, Scopus, and Google Scholar (2000–2025) identified relevant literature using keywords related to oncology nursing, QoL, SNC and patient satisfaction. Inclusion criteria encompassed peer-reviewed studies (quantitative, qualitative, and reviews) involving adult cancer populations and validated assessment instruments. Pediatric and non-nursing studies were excluded. Both QoL and SNC are critical outcomes in cancer care. QoL is an individual’s subjective perception and SNC reflects the patient’s subjective evaluation of how well nursing services align with their expectations, encompassing empathy, competence, and responsiveness. Research consistently demonstrates a strong positive association between high SNC and superior QoL outcomes, particularly through effective pain and symptom management, reducing psychological distress, and fostering patient empowerment. This relationship, however, is moderated by factors such as the patient’s disease stage, the type of care (hospital vs. palliative/home care), and organizational deficits like high nurse workload, which can undermine the delivery of high-quality care. Ultimately, ethical nursing practices are crucial for translating professional duty into positive patient experiences that are favorably linked with both SNC and QoL outcomes for cancer patients.

## 1. Introduction

Cancer remains one of the leading global health challenges, with an increasing incidence and prevalence across all regions of the world [1]. Advances in early detection, chemotherapy, radiotherapy, and targeted biological treatments have improved survival rates; however, the impact of cancer extends far beyond survival statistics. Beyond its biological implications, cancer significantly affects multiple dimensions of patients’ lives, including physical functioning, psychological well-being, social relationships, and overall daily activities [2] that can significantly reduce their overall quality of life (QoL) [3]. The multifaceted burden of cancer encompasses both the acute challenges of intensive clinical treatment and the long-term complexities of survival care, significantly affecting physical functioning, psychological well-being, and social relationships. These necessitate comprehensive approaches that extend beyond direct clinical treatment, emphasizing the need to address holistic aspects of patient care [4]. As the population of cancer survivors grows, nursing care plays a central role in managing this dual burden, delivering personalized support that directly influences patient satisfaction and overall QoL.

The World Health Organization (WHO) defines quality of life (QoL) as “an individual’s perception of their position in life in the context of the culture and value systems in which they live and in relation to their goals, expectations, standards, and concerns” [5] (p. 1403). This definition highlights that QoL is a broad-ranging, multidimensional concept influenced by a complex interplay of factors, including physical health, psychological state, personal beliefs, social relationships, and the individual’s relationship with salient features of their environment. The WHO definition emphasizes the subjective nature of QoL, recognizing that it is specific to each individual’s context and values and is affected not only by health status but also by social and environmental conditions surrounding the person [5].

QoL has emerged as a critical outcome measure in oncological care, serving as a comprehensive indicator that reflects patients’ overall well-being and the effectiveness of therapeutic interventions. QoL encompasses subjective evaluations of physical health, emotional status, social engagement, and symptom burden, thereby providing insight into the patient’s lived experience throughout diagnosis, treatment, and survivorship phases [6]. This is particularly significant with cancer patients’ increasing survival. The integration of QoL assessment into cancer care enables healthcare providers to tailor interventions that mitigate adverse effects, enhance patient-centered outcomes, and ultimately improve survival and rehabilitation trajectories [7].

Nursing care plays a central role in supporting patients through the cancer trajectory. Nurses are responsible not only for technical and clinical interventions but also for providing psychological support, education, and communication that foster trust and comfort [8,9]. Nurses are uniquely positioned to deliver continuous, personalized care that addresses patients’ needs and positively affects their QoL [9]. Furthermore, nurses often act as intermediaries between patients and multidisciplinary teams, amplifying the holistic management of cancer-related challenges [10].

Satisfaction with nursing care (SNC) can be defined as the degree to which patients feel that their expectations and needs regarding nursing services are met by the care they receive. It reflects patients’ subjective evaluation of the quality of nursing care, encompassing factors such as technical competence, empathy, communication, responsiveness, involvement in care, and interpersonal relationships [11,12]. Essentially, patient satisfaction with nursing care gauges how well nursing services align with patients’ values, preferences, and expectations, thereby serving as a measure of the effectiveness and acceptability of nursing interventions in clinical practice [13].

Patient satisfaction is a major indicator of quality care [11]. This concept is considered a crucial indicator of healthcare quality because nursing care often represents the majority of direct patient interaction during hospitalization, influencing not only the patient’s experience but also treatment adherence, perception of health, and emotional resilience and overall health outcomes [14]. The quality of nursing care directly influences patients’ experiences and satisfaction, which are essential contributors to enhanced QoL [15].

While extensive research has explored QoL and SNC independently, a significant gap remains in synthesizing how these constructs interact within the specific organizational and ethical constraints of modern oncology. Most existing literature is cross-sectional, failing to explain the mechanism by which nursing interventions translate into holistic well-being. A narrative review is necessary at this stage to bridge empirical findings with theoretical frameworks offering a multidimensional perspective that accounts for cultural and systemic moderators. While the existing literature and established frameworks in oncology nursing care [4,8], have effectively evaluated independent aspects of oncology nursing care; research often examines QoL [2,3,5,6,7] and SNC as isolated metrics [11,12,13,14,15]. This narrative review addresses this gap by offering a unique, integrated perspective that bridges these empirical findings directly with structural and nursing quality models. The unique value of this synthesis lies in its ability to map how organizational constraints and systemic healthcare inequities influence the relational care processes that dictate holistic patient well-being, thereby providing a comprehensive overview for both clinical practice and macro-level health policy.

This review aims to explore existing evidence on the relationship between QoL and SNC among patients with cancer from a broader public health perspective. The primary research question of this review is: What is the nature of the relationship between QoL and SNC among cancer patients, and how is this relationship influenced by patient, nurse, and organizational factors, as well as systemic healthcare inequities? Understanding this correlation is fundamental for advancing patient-centered models of care, optimizing healthcare workforce planning, and integrating patient-reported outcome measures (PROMs) into national cancer-control strategies to improve population-level health system performance.

## 2. Materials and Methods

This is a comprehensive narrative review, aiming to synthesize and discuss the existing literature on the relationship between quality of life and satisfaction with nursing care in cancer patients. The narrative review approach was selected to allow for a broad, interpretive, and theory-informed discussion of empirical and conceptual evidence. Relevant studies in English were identified through a non-systematic search conducted in September and October 2025 in the databases PubMed, CINAHL, and Scopus. Google Scholar was also utilized as a supplementary source to ensure a comprehensive reach for identifying relevant organizational reports and theoretical papers, thereby facilitating a broader, interpretive synthesis consistent with the narrative review approach. The search was conducted using combinations of the keywords “cancer,” “cancer nursing”, “oncology nursing,” “quality of life,” “patient satisfaction”, and “satisfaction with nursing care”.

Inclusion criteria were studies published between 2000 and 2025 involving adult cancer patients, examining quality of life and/or satisfaction with nursing care, using validated instruments for QoL or satisfaction with nursing care assessment when applicable, and constituting quantitative, qualitative, mixed-methods studies, reviews, or theoretical papers and reports by health organizations relevant to oncology nursing care. Exclusion criteria included studies focusing exclusively on pediatric populations, articles not related to nursing care, non-healthcare-related satisfaction studies, and publications lacking relevance to the review objectives. It should be noted here that a 25-year inclusion window was selected to provide a comprehensive analysis of the relationship between QoL and SNC. This timeframe is necessary because the systematic assessment of QoL in oncology gained significant prominence during this period, transitioning from a secondary observation to a critical patient-reported outcome. Furthermore, many validated instruments specifically tailored for oncology nursing care were developed and refined within this span, allowing for a thematic synthesis that tracks both historical conceptual shifts and recent clinical advancements in nursing interventions.

Study selection was conducted narratively by multiple reviewers from the research team, based on relevance to the review topic, contribution to conceptual understanding, and applicability to oncology nursing practice. The screening of titles, abstracts, and full texts was executed through a collaborative, iterative consensus approach. This strategy ensured that the retrieved articles were qualitatively evaluated to establish a final body of literature with a comprehensive, balanced representation of both historical conceptual shifts and recent clinical advancements.

**Figure 1:**
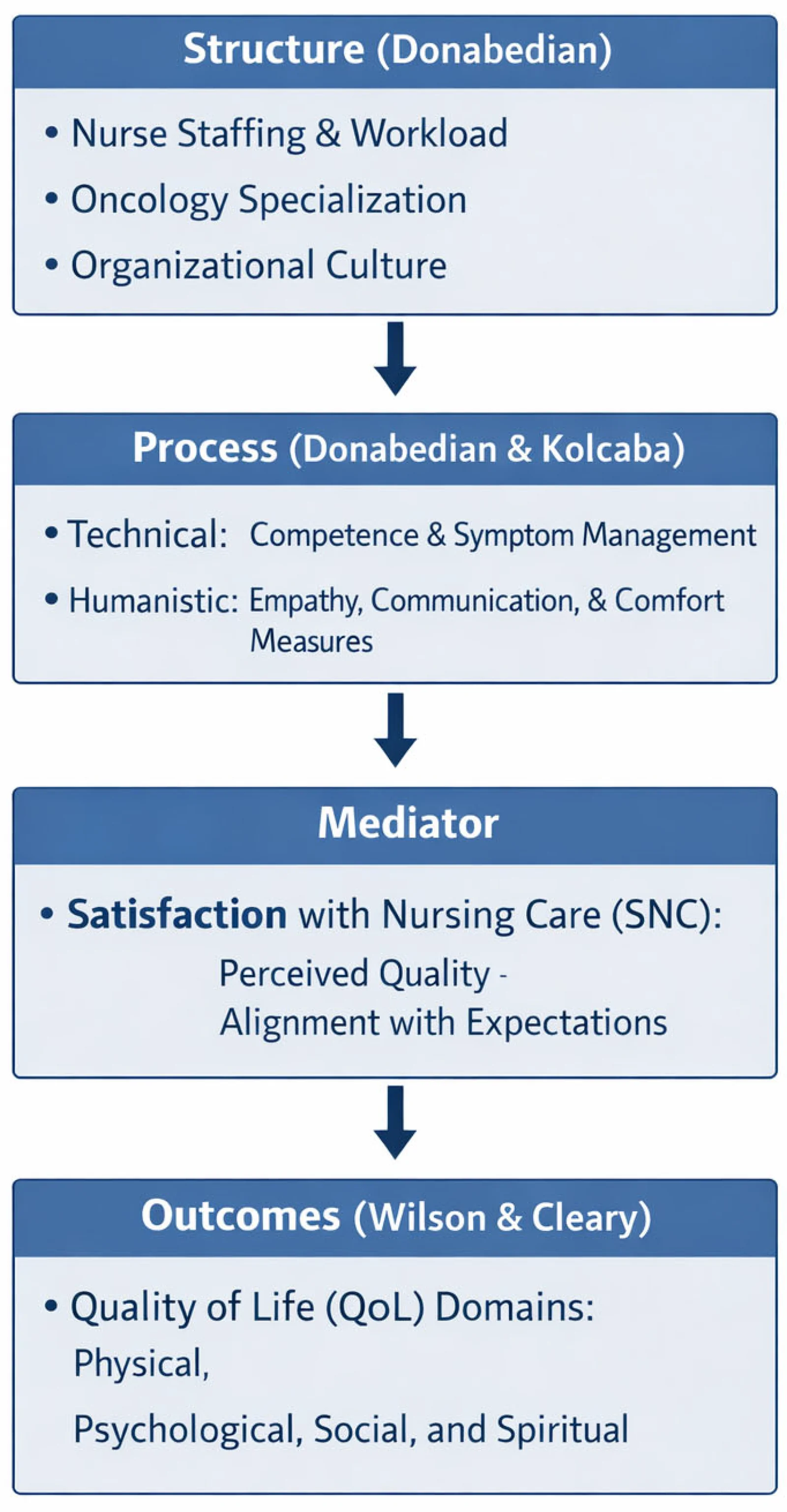
Conceptual figure.

Given the narrative nature of the review, no formal methodological quality or risk-of-bias assessment was performed. However, priority was given to peer-reviewed publications, well-established measurement instruments, and articles published in reputable scientific journals and by recognized health organizations. Finally, Google Gemini (Version Gemini 3.5 flash) was used in the creation of Figure 1.

## 3. Results and Synthesis

The body of literature analyzed in this comprehensive synthesis encompasses a spectrum of peer-reviewed empirical studies, clinical guidelines, and foundational nursing theories exploring the intersecting realities of oncology care. To provide a structured and multi-dimensional overview of the current evidence, the following synthesis is organized into key thematic subsections. First, the clinical impact, progression, and standardized assessment profiles of QoL in oncology are examined. Second, the multi-level determinants and measurement tools of SNC are mapped. Finally, these independent concepts are integrated through established theoretical and conceptual models to systematically analyze the contextual, ethical, and organizational moderators that shape the patient experience across diverse clinical settings.

### 3.1. Effects of Disease Stage and Treatment on QoL

The concept of QoL, particularly in the context of clinical medicine, is often mentioned as health-related quality of life (HRQoL) [16]. It is a highly complex, multidimensional construct that encapsulates an individual’s subjective evaluation of their physical, psychological, social, and spiritual well-being within their lived environment [17].

In oncology, HRQoL serves as a critical patient-reported outcome (PRO) that transcends mere survival metrics, reflecting the holistic impact of the malignant disease and its subsequent therapeutic interventions on the patient’s functional status and existential equilibrium [18]. For cancer patients, the degradation of HRQoL is frequently attributable to a spectrum of debilitating symptoms and psychophysiological sequelae [19], including:

- Somatic Distress: Chronic or acute pain and cancer-related fatigue (CRF), which often persist beyond the cessation of active therapy [20].
- Sleep/Wake Cycle Dysregulation: Significant sleep disturbances (insomnia/hypersomnia) [21].
- Psychological Morbidity: High prevalence of anxiety disorders, depressive symptoms, and existential distress fueled by prognostic uncertainty and the threat to personal integrity [22].
- Functional Decline: Impairment of activities of daily living (ADLs) and instrumental activities of daily living (IADLs), impacting autonomy and dignity [23].

Furthermore, the stage of cancer progression profoundly influences QoL, primarily by dictating the intensity of treatment and the severity of symptoms. In early-stage disease, patients typically have a better baseline QoL, though they experience a significant, acute drop in well-being immediately following diagnosis due to emotional distress and during the course of intensive, potentially curative treatments (e.g., radical surgery, high-dose chemo/radiation) [24,25]. Conversely, patients with advanced or metastatic cancer face a sustained, significant reduction in QoL due to a greater systemic symptom burden (like pain and fatigue), functional decline, and pervasive existential distress [26,27]. For these patients, treatment goals often pivot to palliation and palliative care integration, prioritizing symptom control and maintaining a meaningful QoL, necessitating careful ethical trade-offs between extending survival and minimizing debilitating side effects [27,28,29].

The following table (Table 1) summarizes the key factors across the physical, psychological, social, and spiritual domains that significantly influence the QoL of cancer patients, along with the distinct impacts of disease stage and treatment modalities [4,18,19,20,21,22,23,26,29,30,31,32].

Also, different treatments impose distinct profiles of toxicity and impact on various QoL domains. Generally, more intensive or multimodal therapies are associated with greater, albeit temporary, QoL reduction (Table 2) [3,4,19,24,25,30,33,34,35].

In summary, QoL in oncology care is a dynamic outcome that serves as a vital measure of the patient’s holistic well-being throughout the cancer trajectory, necessitating comprehensive, integrated palliative and supportive care alongside curative efforts [36].

### 3.2. Assessment of QoL in Oncology

The systematic assessment of QoL in oncology gained significant prominence from the 1970s and 1980s onward, propelled by advances in treatment that led to prolonged survival, highlighting the need to evaluate the cost of survival in terms of patient well-being [37]. The standardized measurement of QoL is paramount for clinical trials, treatment monitoring, and supportive care planning. Commonly used, psychometrically validated instruments include:

- WHOQOL: The World Health Organization Quality of Life instrument, most commonly utilized in its abbreviated form, the WHOQOL-BREF, is a widely recognized, cross-culturally developed patient-reported outcome (PRO) measure designed to assess the subjective perception of an individual’s quality of life. The WHOQOL-BREF is generic and evaluates QoL across four principal health domains: physical, psychological, social relationships, and the environment. This 26-item tool allows clinicians to holistically benchmark a patient’s well-being against population norms, making it valuable in large epidemiological studies and clinical trials, including those in oncology [38].
- EORTC QLQ-C30 (European Organization for Research and Treatment of Cancer Quality of Life Questionnaire—Core 30): A widely utilized, cancer-specific instrument comprising a global HRQoL scale, five functional scales (physical, role, emotional, cognitive, social), and nine symptom scales/single items [39]. It also allows for the integration of site-specific modules (e.g., QLQ-BR23 for breast cancer) and palliative care like the EORTC QLQ-C15-PAL, a shortened version of the EORTC QLQ-C30 meant for palliative cancer care patients [40].
- FACT-G (Functional Assessment of Cancer Therapy—General): This instrument measures QoL across four domains: physical well-being (PWB), social/family well-being (SWB), emotional well-being (EWB), and functional well-being (FWB) [41]. It also features numerous validated disease-specific measures (e.g., FACT-L for lung cancer), cancer-specific symptom measures (e.g., FACT Bladder Cancer Symptom Index), treatment-specific measures (e.g., FACT—Bone Marrow Transplantation), symptom-specific measures (e.g., FACT-Anemia), palliative care and spiritual well-being (FACIT-PAL), and many more measures [42].
- SF-36 (Short Form-36): Non-disease-specific instruments like the SF-36 or its shorter iteration, the SF-12, are used to compare the QoL of cancer patients against that of the general population or patients with other chronic conditions. It assesses health-related QoL across eight specific domains: physical functioning, role limitations due to physical health, bodily pain, general health perceptions, vitality, social functioning, role limitations due to emotional problems, and mental health. These domains are aggregated into two main summaries: the Physical Component Summary (PCS) and the Mental Component Summary (MCS) [43].

### 3.3. Satisfaction with Nursing Care in Oncology

#### 3.3.1. Factors Affecting Satisfaction with Nursing Care

Satisfaction with nursing care (SNC) in the oncology setting refers to the patient’s subjective evaluation of the quality and quantity of care they receive from nurses. It is a critical PRO that reflects the analogy between the patient’s expectations and their actual experience of care [44].

In oncology, where patients endure prolonged and intensive treatments, satisfaction goes beyond mere technical competence to encompass humanistic aspects like empathy, emotional support, effective communication, and the nurse’s ability to manage complex physical and psychological symptoms [45]. High patient satisfaction is not just a measure of service quality; it correlates positively with adherence to treatment protocols, reduced psychological distress, and ultimately, better health outcomes [46].

Demographic and disease-specific factors play a significant role in shaping a patient’s satisfaction levels. Studies consistently show that factors such as age and education level can influence expectations and, consequently, perceived satisfaction. For example, older patients might express higher satisfaction due to lower expectations, while younger, more educated patients may be more critical of the care provided [47,48]. Furthermore, the stage of the disease and the intensity of treatment are key variables. Patients in the palliative or end-of-life phase often prioritize comfort, pain management, and emotional presence in their nursing care, leading to higher satisfaction when these needs are met effectively [49]. Conversely, patients undergoing aggressive curative therapy might prioritize technical proficiency and quick resolution of acute side effects [50].

Organizational and systemic factors fundamentally influence the nurses’ ability to deliver satisfactory care. Staffing levels, the nurse-to-patient ratio, and the resulting nurses’ workload are consistently cited as major determinants of patient satisfaction [51]. When nurses are overburdened, the time available for therapeutic communication, teaching, and emotional support—the non-technical aspects highly valued by oncology patients is significantly reduced [52]. Additionally, the organizational culture, including management support, opportunities for specialized oncology training, and effective interprofessional teamwork, indirectly impacts patient satisfaction by ensuring a stable, competent, and cohesive care environment [53]. Poor organizational factors often lead to nurse burnout and reduced compassion, negatively affecting the patient experience [54].

Finally, cultural factors impose a layer of complexity on patient expectations and SNC [55]. A patient’s cultural background, language proficiency, and established health beliefs influence how they perceive and evaluate the care provided [56]. Effective communication, which is central to satisfaction, requires nurses to be culturally competent, meaning they must understand and respect differences in health practices, end-of-life views, and communication styles. For instance, a patient from a culture that emphasizes family involvement in decision-making will likely report low satisfaction if nurses communicate primarily with the patient in isolation. Addressing these diverse factors by implementing patient-centered and culturally sensitive care models is crucial for optimizing satisfaction with nursing care in oncology [57].

Overall, SNC in oncology settings is multidimensional and is shaped by a complex interplay of nurse-related actions, patient characteristics, and the organizational context of care delivery (Table 3) [11,12,13,14,15,47,48,49,50,51,52,53,54,55,56,57].

#### 3.3.2. Assessment of SNC

The assessment of patient SNC is critical for quality improvement, necessitating the use of specialized, psychometrically sound instruments [58]. Among these, the Patient Satisfaction with Nursing Care Quality Questionnaire (PSNCQQ) is frequently utilized to measure the patient’s perspective on the quality of care received [59]. Another widely used tool, particularly adapted from service quality management, is SERVQUAL (Service Quality), which measures the gap between patient expectations and their perceptions across five dimensions of service: reliability, responsiveness, assurance, empathy, and tangibles [60].

Due to the unique, complex, and long-term psychosocial needs of cancer patients, several specialized tools have been developed to capture SNC more accurately in the oncology setting:

- Quality of Oncology Nursing Care Scale (QONCS): This scale is specifically tailored to the cancer care context, focusing on dimensions like responsiveness, individualization, coordination of care, and technical proficiency as perceived by the patient [61].
- Oncology Patients Perceptions of the Quality of Nursing Care Scale (OPPQNCS): Similar to QONCS, this instrument assesses key areas critical to cancer patients, such as the nurse’s ability to provide emotional support and information relevant to their unique illness trajectory [62].
- Service Satisfaction Scale for Cancer Care (SCA): While broader than nursing, this scale includes domains that directly relate to nursing performance, such as care provider manner and skill and the information provided about care, making it highly relevant for evaluating satisfaction in chemotherapy and survivorship clinics [63].
- EORTC IN-PATSAT32 (European Organization for Research and Treatment of Cancer-Inpatient Satisfaction with Care): This tool measures satisfaction with various healthcare providers and the hospital environment for inpatient cancer care. It includes specific subscales for satisfaction with nurses’ technical skills and interpersonal skills [64].

The measurement of SNC reveals that satisfaction is not a static outcome but a dynamic interplay between the quality of the nurses’ interpersonal and professional conduct and the individual patient’s context [65]. Optimizing SNC in oncology thus requires a dual focus: implementing policies that reduce nurse workload and support emotional well-being to enable better performance [66] and fostering a care environment that respects diverse patient expectations and cultural needs to ensure the care delivered is perceived as meaningful and satisfactory [67].

### 3.4. Theoretical and Conceptual Models

Understanding the relationship between QoL and SNC requires a theoretical foundation that connects nursing practice, patient experience, and health outcomes [68]. Several conceptual models and nursing theories provide valuable frameworks for this relationship.

#### 3.4.1. Donabedian’s Model

Donabedian’s classic model conceptualizes healthcare quality through three dimensions: structure, process, and outcome. Within this framework, nursing care represents the process, the interaction between patients and healthcare providers that directly influences outcomes such as QoL and satisfaction. High-quality structures (e.g., staffing levels, training, environment) enable effective processes (e.g., empathy, communication), leading to improved patient outcomes. This model supports the idea that improving nursing care processes can lead to measurable gains in both satisfaction and perceived quality of life [69,70,71,72].

#### 3.4.2. Wilson and Cleary’s Model

The Wilson and Cleary model bridges clinical variables and patient-reported outcomes, linking biological factors, symptom status, functional health, general health perceptions, and overall QoL. Nursing interventions influence several steps in this chain, particularly symptom management and emotional well-being, which in turn affect satisfaction and life quality. This model highlights the multidimensional nature of QoL and underscores how satisfaction with care can mediate the relationship between clinical condition and perceived well-being [73,74].

#### 3.4.3. Kolcaba’s Theory of Comfort

A particularly relevant nursing theory is Kolcaba’s theory of comfort. Kolcaba defines comfort as “the immediate experience of being strengthened through having the needs for relief, ease, and transcendence met in four contexts of experience: physical, psychospiritual, sociocultural, and environmental” [75].

According to this theory, when nurses effectively assess and meet patients’ comfort needs, they enhance both satisfaction (as patients feel cared for, respected, and understood) and QoL (as the physical and emotional distress of illness is alleviated). Comfort thus functions as an intermediary concept linking nursing interventions to patient-centered outcomes [75,76].

For example, a nurse providing timely pain management (physical relief), spiritual support (psychospiritual ease), and family inclusion (sociocultural comfort) contributes to holistic well-being, reinforcing the patient’s perception of quality care. This, in turn, leads to higher satisfaction and improved QoL outcomes. Kolcaba’s framework has been validated in oncology and palliative-care settings, where comfort measures, such as emotional presence, clear communication, and individualized attention, are directly associated with better patient-reported QoL scores [75,76,77].

#### 3.4.4. Combination of Theories

To provide a visually integrated synthesis of these theoretical perspectives, Figure 1 illustrates the proposed conceptual model of the relationship between nursing care and patient outcomes in oncology. In this model, Donabedian’s Structure (e.g., staffing and workload) provides the foundation for the process of care, which encompasses both technical proficiency and the humanistic interventions described by Kolcaba. These processes directly influence SNC, which serves as a critical mediator. Finally, according to the Wilson and Cleary framework, these interactions translate into improved QoL across physical, psychological, social, and spiritual domains. This model emphasizes that QoL is not a static result of medical treatment alone but a dynamic outcome of the organizational environment and the relational quality of the nursing-patient dyad.

### 3.5. Relationship Between QoL and SNC in Oncology

The relationship between QoL and SNC in oncology is essentially linked and mutually reinforcing [47]. SNC is not merely an indicator of service quality; it functions as a mediator and a predictor of QoL. When patients feel their nurses are responsive, communicative, and empathetic, their physical and psychological distress is often attenuated, leading to improved overall QoL. Conversely, poor nursing care can compound the suffering caused by the disease, severely degrading QoL [30,45].

Effective and compassionate nursing care serves as a direct intervention that positively influences multiple domains of a cancer patient’s QoL [78]. Better communication is important, as oncology nurses who effectively explain complex treatment plans, listen actively to fears, and provide timely information reduce anxiety and feelings of uncertainty, thereby improving the psychological and cognitive domains of QoL [79]. Furthermore, oncology nurses are on the front line of pain and symptom management. Cancer pain is a prevalent symptom among cancer patients and can significantly influence their QoL. Effective pain management is crucial to reduce the effects of pain on everyday activities, and cancer nurses play a vital role in every aspect of pain management and enhance the effectiveness of treatment through their knowledge and expertise [80]. Timely and effective symptom control not only alleviates physical distress but also improves functional status and allows patients to engage more fully in social activities, thus boosting physical and social QoL [79]. Nurses also contribute to psychological empowerment by promoting self-efficacy and encouraging patients to participate in care decisions, helping them to regain a sense of control and dignity [81].

Research consistently demonstrates a positive correlation between high SNC and superior QoL outcomes in oncology. Numerous studies show that patients reporting high SNC (especially concerning communication and emotional support) report significantly higher scores in global QoL, emotional functioning, and social functioning, and lower scores for symptoms like fatigue and depression [82,83,84,85]. However, the relation is nuanced; patients undergoing life-saving but highly toxic treatments might report low physical QoL due to side effects, even when their SNC is high [86,87,88]. This demonstrates that while SNC is a strong predictor of psychosocial QoL, its relationship with the purely physical domain can be obscured by acute treatment toxicity.

The positive correlation between QoL and SNC is not static in oncology but is dynamically influenced by many factors, making the relationship context-dependent and complex. Firstly, the dynamics between SNC and QoL vary significantly based on the type of cancer and the care setting. For cancers with high symptom burdens or advanced stages, the nurse’s role in symptom and pain management becomes the paramount driver of QoL, and satisfaction hinges on the effectiveness of these interventions [50,88]. In hospital settings, SNC is often driven by responsiveness and technical competency, leading to immediate symptom resolution [65]. Conversely, in home care, satisfaction is closely tied to the nurse’s teaching ability and support for autonomy, which are key to maintaining long-term independence and QoL [89]. The relationship is perhaps strongest and most crucial in palliative or end-of-life care, where SNC is almost entirely based on meeting psychosocial and existential needs, compassion, dignity maintenance, and comfort care (pain control), defining the patient’s final-stage physical and spiritual QoL [49,90].

Moreover, the relationship is shaped by patient expectations and organizational efficiency. A patient’s prior expectations determine how they evaluate care; those seeking strong emotional support will experience a greater boost in QoL from compassionate nursing and a sharper decline if that support is absent [91]. However, the organizational structure often limits this delivery. High nurse workload and poor staffing ratios reduce the time and energy nurses have for crucial communication and emotional support, thereby weakening the powerful positive link between high-quality relational care and improved QoL [92].

While a positive correlation between SNC and QoL is generally observed, the strength and nature of this relationship vary significantly across contexts. For instance, in acute hospital settings, satisfaction is primarily driven by technical proficiency and rapid symptom relief. In contrast, in palliative and home-care settings, the relationship is more heavily influenced by the nurse’s ability to support patient autonomy and existential comfort. Furthermore, research indicates that while nursing care is a strong predictor of psychosocial well-being, its impact on the physical domain of QoL can be temporarily obscured by the acute toxicity of life-saving treatments.

Finally, ethical and deontological dimensions of nursing care (like beneficence, non-maleficence, confidentiality, equity in care) are central to the relationship between patient satisfaction and QoL in oncology [93]. By ensuring respect for autonomy, holistic comfort, and dignity, nurses translate ethical principles into lived experiences of compassion and trust. An ethically grounded nursing practice, therefore, functions as both a moral duty and a clinical determinant of improved outcomes in cancer care [93,94].

## 4. Discussion

Synthesizing the available literature, a consistent pattern emerges. Nursing care quality and patient satisfaction are integral determinants of QoL in cancer care [95]. The relationship between SNC and QoL is a dynamic system mediated by the alignment of care processes with patient expectations. While technical competence ensures physical safety, the relational and humanistic aspects of nursing, such as empathy, availability, and clear communication, function as the primary drivers for the psychological and spiritual domains of QoL. This relationship is best understood by integrating Donabedian’s framework, which links nursing behaviors to outcomes [69,70,71], with the Wilson and Cleary model, which illustrates how psychosocial processes mediate the pathway from clinical condition to life satisfaction [73]. Furthermore, applying Kolcaba’s theory reveals that QoL is significantly optimized when nursing interventions provide holistic comfort, physical, sociocultural, and existential, to mitigate the burden of disease [75,76,77].

Critically, however, structural deficits such as high nurse workload act as a fundamental barrier, preventing these essential care processes from translating into positive patient outcomes. This workload strain forces an implicit rationing of nursing care, where clinical environments under structural deficits are forced to prioritize immediate technical safety over the time-intensive, relational comfort measures required to optimize a patient’s psychosocial and spiritual well-being [92].

Research consistently identifies a complex array of nurse-related factors that are powerful determinants of patient satisfaction. At the forefront are the nurses’ communication skills, which involve both verbal clarity regarding treatments and prognosis and attentive listening to patient concerns. Equally vital is the nurses’ emotional availability and use of empathy, which is particularly critical in oncology to mitigate distress and foster trust. However, the quality of these interactions is heavily modulated by the organizational environment; high nurse workload and unfavorable nurse-to-patient ratios significantly reduce the time and energy nurses have for effective communication and emotional support, leading to diminished patient satisfaction.

A critical consideration when interpreting the observed relationship between SNC and QoL is the predominance of observational and cross-sectional study designs in the existing literature. While these studies consistently demonstrate positive associations, they do not allow for causal inference or determination of directionality. It remains unclear whether high-quality nursing care directly improves QoL or whether patients with better physical and psychological functioning are more likely to report higher satisfaction with care.

Furthermore, cross-sectional designs are particularly vulnerable to confounding factors such as disease stage, symptom burden, personality traits, care setting, and social support, all of which may independently influence both satisfaction and quality of life. As a result, the strength of the reported associations should be interpreted with caution. Consequently, while the observed positive correlation between SNC and QoL is consistently documented across the literature, readers should view these findings as a conceptual synthesis of reported trends rather than definitive evidence of direct causality. Longitudinal and interventional studies are needed to clarify causal pathways.

### Limitations and Strengths

The strength of this review lies in its comprehensive theoretical and multidimensional approach, which moves beyond clinical data to explore the complex relationship of SNC and QoL in oncology. By integrating empirical evidence with established theoretical frameworks, this review provides a robust conceptual mechanism to explain how nursing interventions translate into holistic patient well-being. Furthermore, the review critically identifies organizational and systemic barriers that affect the success of patient-centered care. This synthesis offers actionable insights for both clinical practice and healthcare administration to improve the lived experience of cancer patients.

Nevertheless, limitations remain. This review is subject to methodological limitations inherent to narrative approaches. As a narrative review, the literature search and selection process was not conducted according to systematic-review protocols, which limits reproducibility and may introduce selection bias. Although multiple databases were searched and priority was given to peer-reviewed and methodologically robust studies, the absence of predefined screening steps and formal quality appraisal may affect the consistency of study inclusion. Also, this review does not provide a quantitative synthesis of findings, such as meta-analysis or formal assessment of effect sizes, which restricts the ability to estimate the magnitude and consistency of observed associations. Rather, the aim was to provide a comprehensive and interpretive overview of the relationship between QoL and SNC in oncology settings, highlighting key themes and gaps in the literature. Future research would benefit from systematic review methodologies and meta-analytic approaches to enhance rigor and support evidence-based conclusions.

Moreover, there is a predominance of observational and cross-sectional studies, without the possibility of establishing causality, and many studies rely on self-reported data, which may introduce bias. Cultural variations also affect perceptions of care; what constitutes “satisfaction” in one context may differ substantially in another. Cultural variations introduce a significant layer of complexity to the perception and assessment of SNC, particularly in oncology. What constitutes “satisfaction” is not universally defined but is profoundly shaped by a patient’s cultural background, established health beliefs, and communication norms. A care practice deemed highly satisfactory in one cultural context may be viewed as inappropriate or even disrespectful in another, making a one-size-fits-all approach to care delivery and quality measurement ineffective [96,97,98]. So, to achieve genuinely patient-centered care in oncology, nurses must move beyond technical competency to embrace cultural sensitivity, recognizing and adapting care practices to the unique, culturally driven expectations of each patient [57].

## 5. Clinical and Administrative Implications

The findings of this review carry significant implications for both clinical nursing practice and healthcare administration. From a clinical perspective, nurses should receive ongoing education in communication, empathy, and psychological support techniques, especially for cancer patients. From an organizational viewpoint, hospital management should monitor SNC and QoL indicators as part of continuous quality-improvement strategies. Integrating PROs into electronic health records can help identify care gaps and tailor interventions [99].

Beyond the micro-level of patient–nurse interactions, QoL and SNC should be conceptualized as core health system performance indicators. From a public health perspective, the relationship between SNC and QoL has important implications for health system performance and population-level monitoring of care quality. Patient-reported outcome measures (PROMs) such as QoL and SNC function as patient-centered performance indicators, complementing traditional metrics such as mortality and morbidity. PROMs provide standardized instruments to operationalize QoL and SNC, enabling their integration into population-level monitoring systems. So, beyond individual care, PROMs facilitate population-level monitoring, allowing policy-makers and health systems to compare patient outcomes across different regions or providers [100].

In line with the WHO framework, these outcomes reflect key dimensions of healthcare quality, including patient-centeredness, effectiveness, and equity. Their integration is particularly relevant within the context of the universal health coverage by WHO, which emphasizes not only access to care but also the delivery of high-quality services that improve population health outcomes [101]. Embedding PROMs into cancer registries, national quality frameworks, and digital health systems enables continuous monitoring of care performance, identification of disparities, and informed policy-making. Therefore, integrating PROMs into routine oncology practice bridges the gap between individual patient experiences and population-level health system evaluation.

In addition, the findings highlight the importance of health workforce planning, as adequate nurse staffing, manageable workloads, and specialized oncology training are key determinants of patient outcomes. Public policies that invest in the nursing workforce and incorporate patient-reported indicators into quality-monitoring frameworks can guide resource allocation, identify care gaps, and strengthen value-based cancer care at the population level [102].

Policy-makers should also recognize nursing care quality as a key component of national cancer control strategies. Investment in adequate staffing ratios, supportive work environments, and professional development enhances not only nurse satisfaction but also patient outcomes [103]. On a macro-structural level, the interplay between SNC and QoL is profoundly shaped by global health inequalities. While this review establishes a positive correlation between nursing care and patient well-being, this relationship is frequently undermined in low- and middle-income countries by significant oncology nursing workforce shortages and limited access to specialized training. Unlike high-income settings where quality frameworks are well-integrated into digital health systems, resource-constrained environments often face nursing rationing, where high workloads prevent the delivery of the humanistic, relational care necessary to boost patient QoL [104]. Furthermore, while global health quality frameworks emphasize patient-centeredness, international disparities in healthcare funding mean that for many cancer patients globally, satisfaction is dictated more by the availability of basic clinical safety than by the holistic support found in integrated care models. Addressing these population-level disparities is essential for translating the ethical duty of nursing into equitable QoL outcomes worldwide [102].

Overall, promoting a culture of compassionate, evidence-based and patient-centered nursing care is essential for improving the lived experience of cancer patients.

## 6. Future Research Directions

Future research should transition from cross-sectional designs toward longitudinal and interventional studies to establish the causal impact of nursing care on QoL over the cancer trajectory. Specifically, investigations should focus on developing and validating culturally sensitive instruments for assessing SNC and QoL across diverse oncology populations. Interventional studies are also needed to evaluate how specific nursing programs, such as communication training or mindfulness-based support, directly influence SNC and QoL outcomes. Additionally, studies should explore the integration of digital real-time feedback platforms to monitor SNC and examine the organizational thresholds where nurse workload begins to compromise the delivery of compassionate, relational care.

In addition, qualitative explorations can provide deeper insight into patient experiences, particularly in palliative and home care settings. Cross-cultural comparisons would help distinguish universal from culture-specific dimensions of SNC. Finally, integrating technology, including digital feedback platforms, could enable real-time monitoring of patient satisfaction and help tailor nursing interventions more effectively.

## 7. Conclusions

Quality of life and satisfaction with nursing care exhibit a highly interconnected relationship within the oncology care experience. Beyond medical treatment, the quality of nursing interactions, characterized by empathy, competence, and respect, is consistently associated with how patients cope with illness, adhere to therapy, and perceive their journey. Recognizing these observed associations reinforces the value of holistic, patient-centered nursing practice. By investing in professional education, supportive organizational structures, and ethical awareness, healthcare systems can foster both patient satisfaction and improved quality of life for individuals living with cancer.

From a public health perspective, prioritizing high-quality oncology nursing serves as a scalable intervention that reduces the societal burden of cancer by improving treatment adherence and long-term survivorship across diverse populations. Furthermore, by integrating patient-centered care into systemic policy, healthcare authorities can more effectively address health disparities and ensure equitable access to the psychological and physical support necessary for community-wide well-being.

## Figures and Tables

**Table 1:** Factors influencing QoL in oncology [4,18,19,20,21,22,23,24,26,29,30,31,32].

Domain	Key Influencing Factors	Effect of Disease Stage and Treatment
1. Physical [4,18,19,20,21,22,23,24]	Symptom burden: Severity of pain, CRF, nausea, dyspnea. Functional status: Ability to perform ADLs and maintain physical activity (performance status). Treatment-related changes: Alopecia, weight changes, body image alteration, sexual dysfunction.	Early stage: QoL often dips sharply during intensive treatment (surgery, high-dose CT/RT) but may recover fully. Advanced stage: Chronic, high symptom burden leads to persistent low functional QoL. Chemotherapy: Acute, systemic functional decline (e.g., neuropathy, myelosuppression). Surgery/RT: Localized functional loss or disfigurement, often followed by recovery.
2. Psychological (emotional/cognitive) [19,22,30,31,32]	Emotional distress: High prevalence of anxiety, depression, and overall emotional distress. Cognitive function: Presence of CRCI, affecting memory and concentration. Fear and uncertainty: FoR or disease progression; prognostic uncertainty. Coping mechanisms: The patient’s psychological resilience and coping strategies.	Across all stages: Diagnosis and prognosis create significant psychological distress. Chemotherapy/hormonal therapy: May induce or exacerbate mood disorders and CRCI. Survivorship: FoR remains a dominant negative psychological factor even after curative intent treatment.
3. Social (interpersonal and role functioning) [4,18,30,32]	Social support: Quality and availability of family and social network support. Role functioning: Loss of the ability to maintain work, family, and social roles. Financial toxicity: Economic strain from treatment costs, lost wages, and reduced productivity. Caregiver burden: Patient’s perception of the burden placed on family caregivers.	All stages: Disease and treatment can cause temporary or permanent social isolation and role dysfunction. Advanced stage: Increased dependency on others reduces autonomy and social function. Treatment breaks: May allow for temporary improvements in social engagement.
4. Spiritual (existential well-being) [26,27,28,29]	Meaning and purpose: Questioning the meaning of life, suffering, and the disease experience. Spiritual distress: Feelings of isolation, loss of faith, or conflict with belief systems. Hope: The ability to maintain hope, acceptance, and a sense of transcendence. Religious/spiritual coping: Use of faith or spirituality as a coping resource.	Advanced stage: Spiritual and existential distress often peak when the prognosis is poor and curative options are exhausted. Palliative care: Integrated spiritual support can significantly enhance QoL and reduce distress toward the end of life.

Abbreviations: CT: chemotherapy; CRCI: cancer-related cognitive impairment; CRF: cancer-related fatigue; FoR: fear of recurrence; RT: radiotherapy; QoL: quality of life.

**Table 2:** Impact of cancer treatments on QoL [3,4,19,24,25,30,33,34,35,36].

Treatment	Primary Impact on QoL Domains	Sequelae
CT [3,19,24,30,33]	Physical: fatigue, nausea/vomiting, mucositis, myelosuppression, neuropathy. Psychological: CRCI, Anxiety.	QoL often decreases acutely during cycles but may improve between cycles or significantly improve if the CT is effective at reducing tumor burden/symptoms.
RT [33,34]	Physical: Localized fatigue, skin reactions, organ-specific dysfunction, e.g., pneumonitis, proctitis.	QoL reduction is often localized and may be less systemic than CT, but long-term sequelae (e.g., fibrosis, chronic pain) can be significant.
Surgery [4,19,25,30]	Physical: Pain, functional loss, recovery time. Psychological and social: Distress, body image alteration, role changes.	QoL reduction is sharp post-procedure but tends to recover quickly. Long-term impact depends on the degree of functional or aesthetic impairment (e.g., lymphedema, stoma).
Immunotherapy/targeted therapy [35,36]	Varied, often physical (specific immune-related adverse events, e.g., colitis, thyroiditis) but generally better tolerated than conventional CT.	QoL profile is often more favorable, but the chronic nature of some side effects requires long-term management.

Abbreviations: CT: chemotherapy; CRCI: cancer-related cognitive impairment; RT: radiotherapy; QoL: quality of life.

**Table 3:** Factors influencing SNC in oncology [11,12,13,14,15,47,48,49,50,51,52,53,54,55,56,57].

Factor	Key Influencing Elements	Impact on SNC
Nurse-related [11,12,13,44,45,46]	Communication skills: Clarity in explaining treatment, active listening, and providing information. Emotional availability/empathy: Showing compassion, reducing distress, and building trust. Technical competence: Proficiency in performing clinical tasks (e.g., administering chemotherapy, managing central lines). Responsiveness: Timeliness in responding to calls, pain, and urgent needs.	Directly increases: When nurses exhibit strong communication and empathy, patients feel valued and understood. Directly increases: Patients feel safe and confident when technical care is perceived as high quality.
Patient-related [47,48,49,50,55,56,57]	Patient expectations: Prior experiences, cultural norms, and media influence the benchmark against which care is measured. Cultural background: Need for culturally sensitive care; language barriers reduce comprehension and trust. Severity of illness/symptom burden: Patients with severe pain or distress prioritize rapid response and effective symptom management. Age and education: May influence critical assessment skills and the ability to articulate needs.	Modulates satisfaction: Mismatch between high expectations and perceived care leads to dissatisfaction. Influences perception: Care that ignores cultural norms or involves language barriers leads to low satisfaction. Shifts priorities: Satisfaction becomes highly dependent on meeting basic comfort needs.
Organizational [51,52,53,54]	Nurse workload/staffing ratios: High workload reduces the time nurses have for non-technical, humanistic care. Organizational culture: Management support, inter-professional teamwork, and specialized oncology training. Physical environment: Cleanliness, comfort, and noise levels of the oncology unit.	Indirectly decreases: A high workload leads to rushed care, lower emotional availability, and burnout. Indirectly increases: A supportive, well-trained team provides consistent, high-quality care. Modulates comfort: A poor environment detracts from the overall positive experience of care.

## Data Availability

No new data were created or analyzed in this study. Data sharing is not applicable to this article.

## Abbreviations

The following abbreviations are used in this manuscript:

ADLs	Activities of daily living
Chemo/RT	Chemotherapy and radiation therapy
CRCI	Cancer-related cognitive impairment
CRF	Cancer-related fatigue
CT	Chemotherapy
ECOG	Eastern Cooperative Oncology Group Performance Status
FoR	Fear of recurrence
HRQoL	Health-related quality of life
PRO	Patient-reported outcome
PROM	Patient-reported outcome measures
QoL	Quality of life
RT	Radiation therapy
SNC	Satisfaction with nursing care
WHO	World Health Organization
